# Optical Gaps of Ionic Materials from GW/BSE-in-DFT and CC2-in-DFT

**DOI:** 10.1021/acs.jctc.4c00819

**Published:** 2024-10-17

**Authors:** Manas Sharma, Marek Sierka

**Affiliations:** Otto Schott Institute of Materials Research, Friedrich Schiller Unversity Jena, Löbdergraben 32, 07743 Jena, Germany

## Abstract

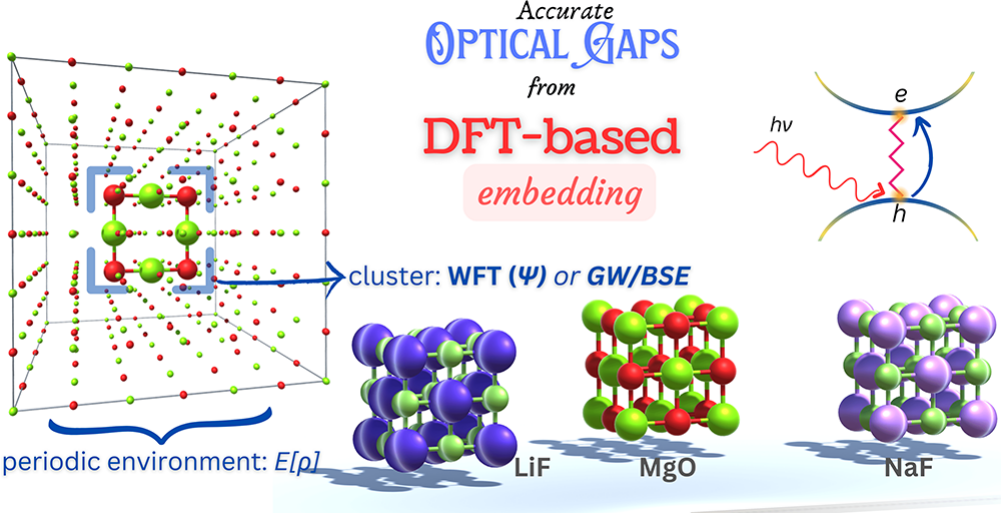

This work presents a density functional theory (DFT)-based embedding technique for the calculation of optical gaps in ionic solids. The approach partitions the supercell of the ionic solid and embeds a small molecule-like cluster in a periodic environment using a cluster-in-periodic embedding method. The environment is treated with DFT, and its influence on the cluster is captured by a DFT-based embedding potential. The optical gap is estimated as the lowest singlet excitation energy of the embedded cluster, obtained using a wave function theory method: second-order approximate coupled-cluster singles and doubles (CC2), and a many-body perturbation theory method: GW approximation combined with the Bethe–Salpeter equation (GW/BSE). The calculated excitation energies are benchmarked against the periodic GW/BSE values, equation-of-motion coupled-cluster singles and doubles (EOM-CCSD) results, and experiments. Both CC2-in-DFT and GW/BSE-in-DFT deliver excitation energies that are in good agreement with experimental values for several ionic solids (MgO, CaO, LiF, NaF, KF, and LiCl) while incurring negligible computational costs. Notably, GW/BSE-in-DFT exhibits remarkable accuracy with a mean absolute error (MAE) of just 0.38 eV with respect to experiments, demonstrating the effectiveness of the embedding strategy. In addition, the versatility of the method is highlighted by investigating the optical gap of a 2D MgCl_2_ system and the excitation energy of an oxygen vacancy in MgO, with results in good agreement with reported values.

## 1 Introduction

Accurate predictions of the optical properties of ionic solids are a critical challenge in computational materials science due to the importance of these materials in electronic and optoelectronic applications. Density functional theory (DFT) is popularly used for studying the ground-state properties of materials, owing to its balance of accuracy and computational efficiency.^1−6^ However, its ability to predict excited-state properties, such as optical gaps, is often disappointing. Unlike the fundamental gap which is defined by charged excitations, the optical gap is defined by a neutral excitation between the ground state and the lowest dipole-allowed excited state. Time-dependent DFT (TDDFT)^7^ has difficulty accurately describing neutral excitations, unless hybrid functionals or frequency-dependent exchange-correlation kernels are employed.^8−14^ On the other hand, Green’s function-based many-body perturbation theory methods, such as the GW approximation^15−17^ combined with the Bethe–Salpeter equation^14,18^ (GW/BSE), provide more accurate results for excited-state properties but come with a high computational cost and scale as *O*(*N*^4^) with system size. Additionally, most GW/BSE implementations employ a plane-wave basis, making it costly to study excitonic properties in 2D systems due to the requirement for a large vacuum region.

Many popular ground-state wave function theory (WFT) methods from quantum chemistry, such as the second-order perturbative Mo̷ller–Plesset theory (MP2) and coupled-cluster theory, have been adapted for periodic systems over the last few decades.^19−25^ Recently, equation-of-motion coupled-cluster theory with single and double excitations (EOM-CCSD) has been shown to perform exceptionally well for excited-state properties of solids, including band gaps and optical spectra,^26−30^ emerging as a lucrative alternative to TDDFT or GW/BSE. Despite its accuracy, EOM-CCSD also has a high computational cost and unfavorable *O*(*N*_*k*_^4^*N*_o_^2^*N*_v_^4^) scaling, where *N*_*k*_ is the number of *k*-points, *N*_o_ is the number of occupied orbitals and *N*_v_ is the number of virtual orbitals. This makes EOM-CCSD even more computationally demanding than the already expensive GW/BSE method. Therefore, the applicability of EOM-CCSD is limited to small systems or necessitates substantial computational resources for larger systems.

Embedding techniques offer a viable solution to alleviate the computational cost of periodic GW/BSE and EOM-CCSD methods by partitioning the system into an active region and an environment. This approach allows different levels of theory to be applied to different parts of the system, significantly reducing computational costs while maintaining high accuracy. This manuscript explores a DFT-based embedding approach, which comes under the class of QM/QM embedding or quantum embedding schemes where both subsystems are treated at the quantum mechanical level but with varying approximations. Since electron density is the central quantity in DFT, it serves as the basis for dividing the system into active and environment subsystems. The environment is treated at the DFT level, while the active subsystem may be treated using a higher level method like GW/BSE or a correlated WFT method enabling GW/BSE-in-DFT or WFT-in-DFT calculations, respectively. The influence of the environment on the active subsystem is manifested via an embedding potential. One of the most notable approaches to realize DFT-based embedding is the frozen density embedding (FDE) technique.^31,32^ In FDE, for a given frozen environment density, the density of the embedded active subsystem is obtained by minimizing the total energy bifunctional with respect to the active subsystem density, in the presence of a DFT-based embedding potential.

The coupling of WFT methods to DFT-based embedding (WFT-in-DFT) has been shown to improve the description of both the ground and excited states of the embedded subsystem.^32−35^ In particular, the second-order approximate coupled-cluster singles and doubles (CC2) method^36,37^ combined with DFT-based embedding (CC2-in-DFT) has been used to compute the excitation energies of complex chemical systems efficiently and accurately.^38−46^ Moreover, it is also possible to treat both the active and environment subsystems using CC2 allowing one to perform CC2-in-CC2 calculations, as shown by Höfener and Visscher.^47,48^ While WFT-in-DFT approaches are now commonplace, recent developments reveal that GW/BSE can also integrate well with DFT-based embedding techniques.^49−52^

This work reports an implementation of GW/BSE-in-DFT and WFT-in-DFT embedding for the calculation of excitation energies within the TURBOMOLE program package^53−55^ that employs Gaussian-type orbitals (GTOs) as basis functions for both molecular and periodic systems.^56−58^ This allows for a straightforward computation of the embedding potential for cluster-in-periodic embedding, enabling easy coupling to existing correlated WFT methods within TURBOMOLE or external quantum chemistry software. Building upon a previous study by the same authors,^59^ where a cluster-in-periodic implementation was leveraged for the calculation of WFT-corrected adsorption energy of the H_2_–H_10_ toy model, the current research investigates the use of cluster (GW/BSE or CC2)-in-periodic (DFT) embedding for calculating the optical gaps of ionic materials. The supercell of the ionic material is partitioned into a small molecule-like cluster (active region) and a periodic environment which is described by DFT using local density approximation (LDA) or generalized-gradient approximation (GGA) for the exchange–correlation term. The lowest-lying singlet excitation energy of the cluster embedded in a periodic environment (active subsystem) is calculated using GW/BSE as well as CC2. This lowest excitation energy is considered as an approximation to the optical gap. Using this DFT-based embedding approach, the optical gaps of three-dimensional (3D) ionic solids (MgO, CaO, LiF, NaF, KF, and LiCl), as well as two-dimensional (2D) MgCl_2_, are computed accurately. Additionally, the first excitation energy of an oxygen vacancy in a 3D MgO (neutral *F*-center) is also calculated. The calculated excitation energies are validated against periodic EOM-CCSD results, GW/BSE benchmarks from the literature, and experimental values. The proposed embedding strategy achieves good agreement with high-level reference data at a fraction of the computational cost.

The manuscript is organized as follows. Section 2 provides the theory of frozen density embedding (FDE), along with its extension to correlated WFT methods as well as GW/BSE for the calculation of excitation energy, and the implementation details. The computational details are presented in Section 3. In Section 4, the results obtained using GW/BSE-in-DFT and CC2-in-DFT are provided and discussed in detail, with focus on the effect of cluster size and dependence on the XC functional used for the embedding potential. Finally, the results are summarized and an outlook on future research is provided in Section 5.

## 2 Theory and Implementation Details

### 2.1 Frozen Density Embedding

The theoretical framework for FDE was first put forth by Wesołowski and Warshel in their seminal paper^31^ based on prior works by Senatore and Subbaswamy,^60^ and Cortona.^61^ FDE involves dividing the total system into an active (subsystem of interest) and an environment subsystem such that the total electronic density is given by the sum of the subsystem densities
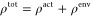
1Following the partitioning, the total energy of the system is given as a bifunctional of the subsystem densities
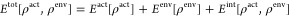
2

The subsystem energies (*E*^i^, where i represents either “act” or “env”), in the above expression, are essentially their individual KS-DFT energies. The interaction energy *E*^int^ between subsystems in eq 2 is given by
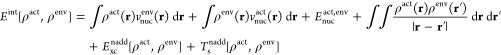
3where *v*_nuc_^act^ and *v*_nuc_^env^ are the electrostatic potentials due to the nuclei in the active and environment subsystems, respectively, and *E*_nuc_^act,env^ is the nuclear repulsion energy between the subsystems’ nuclei. The fourth term represents the electrostatic Coulomb repulsion between electrons of the two subsystems. The last two terms *E*_xc_^nadd^[ρ^act^, ρ^env^] and *T*_s_^nadd^[ρ^act^, ρ^env^] are the nonadditive XC and kinetic energies, respectively, defined as
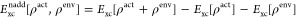
4
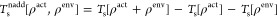
5

The density of the embedded active subsystem ρ^act^ in the presence of a given frozen non-negative ρ^env^ can be determined by minimizing the total energy bifunctional (eq 2) with respect to ρ^act^ while keeping ρ^env^ fixed under the constraint that ρ^act^ is *v*_s_-representable (i.e., there exists a noninteracting reference system leading to the same non-negative ρ^act^ with the same number of electrons as in the active subsystem). This minimization leads to a set of KS-like equations also known as KS equations with constrained electron density (KSCED equations)
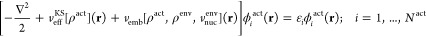
6where *v*_eff_^KS^[ρ^act^](**r**) is the regular effective KS potential for the active subsystem that consists of the nuclear, Hartree and XC potential terms that would usually be present in the KS-DFT calculation of the isolated active subsystem. The effect of the environment on the active subsystem is captured through the embedding potential *v*_emb_ term in eq 6 that depends on subsystem densities ρ^act^ and ρ^env^, as well as the nuclear potential due to the environment *v*_nuc_^env^. It is defined as
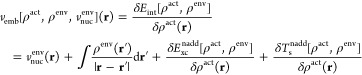
7The first two terms correspond to the potential due to the environment’s nuclei and electrons, respectively. The embedding potential also includes the nonadditive XC potential and the nonadditive kinetic potential (naddKP) terms, which are obtained as the functional derivatives of *E*_xc_^nadd^ (eq 4) and *T*_s_^nadd^ (eq 5), respectively:
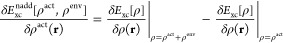
8
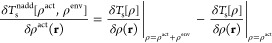
9The naddKP term enforces the Pauli exclusion principle between electrons in the active and environment subsystems. Since the KS orbitals of the total system are unknown and only the total density ρ^tot^ is available, naddKP (eq 9) and *T*_s_^nadd^ (eq 5) must be evaluated using approximate orbital-free kinetic energy density functionals (KEDFs) in practical applications. This limits the applicability of FDE to systems with weakly overlapping subsystem densities.^62,63^

If the exact KEDF was known, the embedding potential and FDE scheme should accurately reproduce the total KS-DFT density ρ^tot^, assuming ρ^env^(**r**) ≥ 0 and that the active subsystem density is *v*_s_-representable. This requires that the environment density never exceeds the total density at any point (∀**r**; ρ^tot^(**r**) ≥ ρ^env^(**r**)),^64^ ensuring ρ^act^ = ρ^tot^ – ρ^env^ is non-negative everywhere. However, achieving this in practice is challenging as many ρ^env^ choices can lead to negative areas in ρ^act^, limiting the selection of permissible frozen densities.^41,65^

#### 2.1.1 WFT-in-DFT for Excitation Energy

In this work, the WFT-in-DFT excitation energy of the active subsystem is computed as the difference between the excited state and ground state energy of the active subsystem:
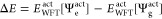
10where *E*_WFT_^act^[Ψ_g_^act^] and *E*_WFT_^act^[Ψ_e_^act^] are the energies of the ground and excited states of the active subsystem, obtained in the presence of embedding potential.

In principle, a different embedding potential should be used for the excited state and ground state energy calculations. This is because the embedding potential is a functional of both active and environment subsystem densities, and the environment density will change in response to the excitation in the active subsystem. If the embedding potential is updated using the polarized subsystem densities then it is termed as being state-specific.^66^ As an approximation, the polarization of the environment may be neglected by keeping the environment density unchanged, especially in cases where the excitation is localized to the active subsystem. This is a reasonable assumption for subsystems with uncoupled or weakly coupled excitations. In this case, the difference in the excited and ground state embedding potential is only due to the different excited and ground state density of the active subsystem. The calculation of WFT-in-DFT excitation energies can be further simplified by simply using the state-independent ground state embedding potential for the calculation of excited and ground state energies of the active subsystem. This is the approach employed in this work and is also referred to as linearized FDET approach in literature.^67,68^ One additional approximation is made by not including the contribution of the embedding potential ∫ρ_g/e_^act^*v*_emb_ d**r** or tr[**D**_WFT_^act^**V**_emb_[**D**_DFT_^act^, **D**_DFT_^env^]] to the ground state and excited state energies, where **D**_WFT/DFT_^act/env^ are WFT and DFT density matrices of the subsystems and **V**_emb_ is the matrix representation of the embedding potential. Including this contribution would have amounted to a first-order correction to the energy of the embedded active subsystem’s wave function.^69^

#### 2.1.2 GW/BSE-in-DFT for Excitation Energy

In the GW/BSE approach, the GW approximation, which calculates the quasiparticle energies by including electron–electron interactions beyond the mean-field level, is combined with BSE to capture electron–hole interactions responsible for excitations. This combination allows for the accurate determination of optical properties in complex systems.

DFT-based embedding allows to use the expensive GW/BSE method only for the excitation energies of the active subsystem, enabling GW/BSE-in-DFT calculations. The embedding potential generated from the surrounding environment acts as an external potential that influences the subsystem, allowing for the effects of the larger environment to be incorporated without explicitly treating it at the GW/BSE level. Once the molecular orbitals and energies of the active subsystem are determined from DFT-in-DFT embedding calculation, they can be used as starting point for the subsequent GW and BSE calculation.

While DFT-in-DFT can be performed exactly (in principle), provided the exact KEDF is known, GW/BSE excitation energies are not expected to exactly match those obtained from a full GW/BSE calculation. The exchange part of the self-energy (Σ_x_) and the correlation part (Σ_c_) are both impacted by the reduction in the number of occupied states and the changes in the molecular orbitals themselves. For example, in the exchange part, Σ_x_ involves a summation over the occupied orbitals, which is truncated in the embedded calculation. This truncation, combined with the changes in the molecular orbitals due to the embedding, affects the quasiparticle energies. Specifically, while Σ_x_ itself only depends on the occupied states, its influence extends to both occupied and unoccupied states, thereby altering the energy gaps predicted by the calculation.

The correlation part (Σ_c_) is even more complex, as it involves the frequency-dependent screened Coulomb interaction, which is modified by the embedding. The screened interaction is determined by the irreducible polarizability, which, in an embedded system, is limited to transitions within the active region. Consequently, the absence of screening from the inactive region typically results in weaker contributions to Σ_c_, leading to larger quasiparticle gaps when compared to full-system calculations.^52^

For further technical details and mathematical formulations related to GW/BSE-in-DFT, the reader is referred to the work by Tölle et al.^49,50^ and other foundational studies in this area.^51,52^

#### 2.1.3 Implementation Details of Cluster-in-Periodic WFT-in-DFT

The strategy for embedding a molecule or a molecule-like cluster as the active subsystem within a periodic environment adopted in this work is similar to the one used in a previous publication (denoted as Method 2) by the same authors^59^ and draws inspiration from prior works by Carter and co-workers.^70−72^ To begin with, the density of the total system ρ^tot^ and the density of the isolated active subsystem ρ_iso_^act^ are obtained using a lower level of theory such as DFT with an LDA/GGA functional. Subsequently, the environment density ρ^env^ is approximated as .

The active subsystem density is then relaxed in the presence of a frozen KEDF-based embedding potential computed using fixed  and ρ_iso_^act^ as the environment and active subsystem densities, respectively. The decision to keep the embedding potential fixed is based on previous experiences where updating the potential with the active subsystem density (ρ^act^) during the DFT-in-DFT embedding caused convergence problems, as documented in refs (72) and (73).

The procedure outlined above, makes the implementation of cluster-in-periodic embedding in TURBOMOLE quite straightforward, since TURBOMOLE computes the KS matrix of periodic systems in real space.^58^ For detailed equations, the readers are referred to ref (59).

WFT-in-DFT excitation energies are calculated by first invoking an HF calculation with the embedding potential added to the HF Hamiltonian (Fock matrix) as a constant term. The CC2-in-DFT excitation energies of the active subsystem can then be calculated using the HF reference orbitals of the embedded active subsystem. As also mentioned before, the same embedding potential is used for ground and excited state avoiding the need for updating the embedding potential.

Similarly, GW/BSE-in-DFT calculations are carried out by using the embedded KS orbitals of the active subsystem to calculate the GW-in-DFT quasiparticle energies, which are then used to solve the BSE for the excitation energy evaluation.

Finally, it is important to mention that the approximation for the environment density employed above cannot be guaranteed to be non-negative everywhere, which may result in additional errors.

## 3 Computational Method and Details

All the calculations employ pob-TZVP-rev2 basis sets throughout.^74^ DFT-based calculations employ density fitting, using a universal auxiliary basis set,^75^ for the evaluation of the Coulomb term. The calculations are carried out with stringent convergence criteria, achieving energy convergence within 1.0 × 10^–7^ a.u. and root-mean-square convergence of density matrix elements within 10^–7^ during the SCF cycle. DFT-based calculations are carried out using the riper module,^56−58^ while the ricc2 module^37^ of TURBOMOLE is used for CC2 and CC2-in-DFT calculations. GW/BSE and GW/BSE-in-DFT calculations are performed using the escf module.^76−78^ Note: only the single-shot G_0_W_0_ is used for the calculation of quasiparticle eigenvalues, the results of which are used as input for solving the BSE.

**Figure 1:**
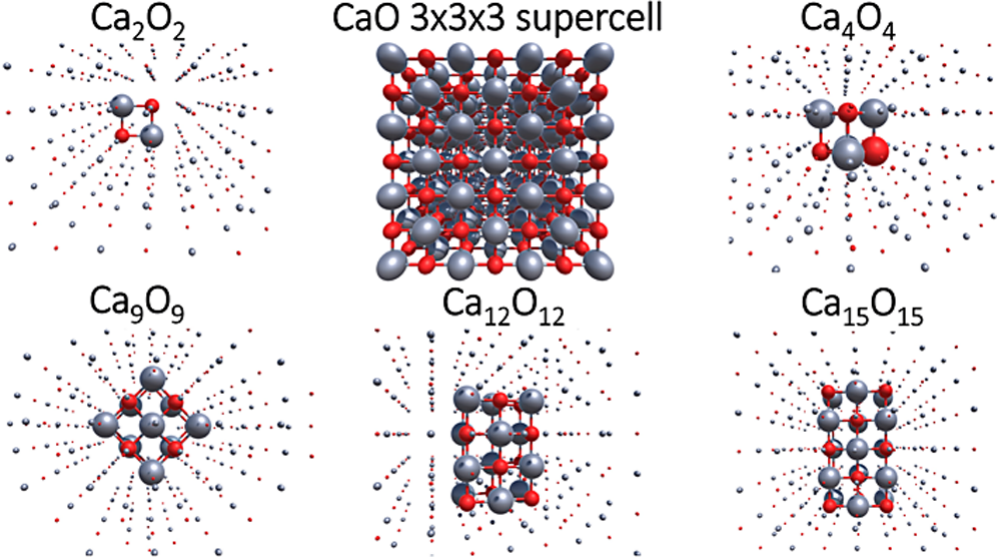
Illustration of the 3 × 3 × 3 CaO supercell and the stoichiometric CaO clusters (larger atoms) of different sizes along with their periodic environment represented with smaller atoms (gray: Ca and red: O).

For the calculation of optical gaps (first singlet excitation energy) of the ionic solids, conventional unit cells (rock-salt phase) with the experimental lattice constants are utilized: MgO with a lattice constant of 4.19 Å,^79,80^ CaO at 4.81 Å,^81,82^ LiF at 3.99 Å,^83−85^ NaF at 4.62 Å,^86,87^ KF at 5.35 Å,^86,87^ and LiCl at 5.13 Å. Starting from the conventional cubic unit cells of these materials, a 3 × 3 × 3 supercell, comprising 216 atoms, is created. Subsequently, clusters of varying sizes are modeled following the principles of neutrality, stoichiometry, and coordination.^88^ Stoichiometric clusters A_*n*_X_*n*_ (where AX is MgO, CaO, LiF, NaF, KF, or LiCl; *n* = 2, 4, 9, 12, 15) are carved out from the supercell, ensuring that they maintain charge neutrality. These clusters act as the molecular active subsystem for embedding while the remaining atoms of the supercell act as the periodic environment, which is treated at the DFT level. Figure 1 illustrates the structures of the clusters of different sizes and the supercell they are carved from, using CaO as an example. The structures for other ionic solids are similar, differing only in their lattice constants of the conventional cell. For reference, periodic EOM-CCSD excitation energies are calculated using PySCF.^89,90^ Additionally, the first singlet excitation energy is also calculated for the 3D ionic solids using the periodic TDDFT implementation in PySCF for more context. The conventional unit cell is utilized for both EOM-CCSD and TDDFT calculations and the Brillouin zone is sampled with 3 × 3 × 3 *k*-points. Starting from a Hartree–Fock reference, the CCSD and EOM-CCSD calculations employ frozen orbitals, and the number of correlated occupied and virtual orbitals (*N*_o_, *N*_v_) for the different systems are MgO (16, 13), CaO (16, 16), LiF (16, 16), NaF (12, 16), KF (15, 15), and LiCl (16, 13). Note that the results may not be fully converged with respect to the number of correlated occupied and virtual orbitals, as these were constrained by the maximum limits of the available computational resources (64 cores; 512 GB memory; 3 TB solid-state drive storage). Furthermore, a 3 × 3 × 3 *k*-grid may not be enough for EOM-CCSD calculations to be converged and would therefore contain finite-size effects (see refs (27) and (29)).

**Figure 2:**
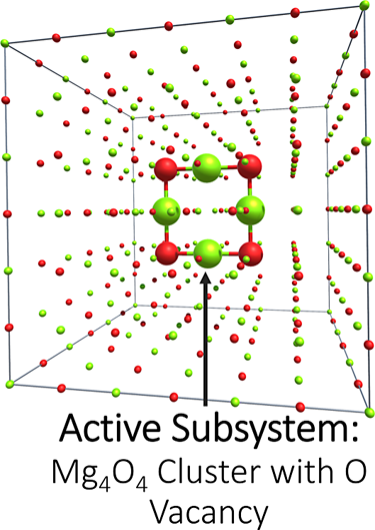
Mg_4_O_4_ cluster with an O vacancy at the center, carved out from the 3 × 3 × 3 supercell of MgO. The active subsystem is denoted by the larger atoms, while the smaller atoms correspond to the environment (green: Mg and red: O).

For the study of the excitation energy of the oxygen vacancy in MgO, an oxygen atom is deleted from the center of the 3 × 3 × 3 supercell of MgO, and the surrounding Mg_4_O_4_ cluster is considered as the active subsystem. The structure is shown in Figure 2.

**Figure 3:**
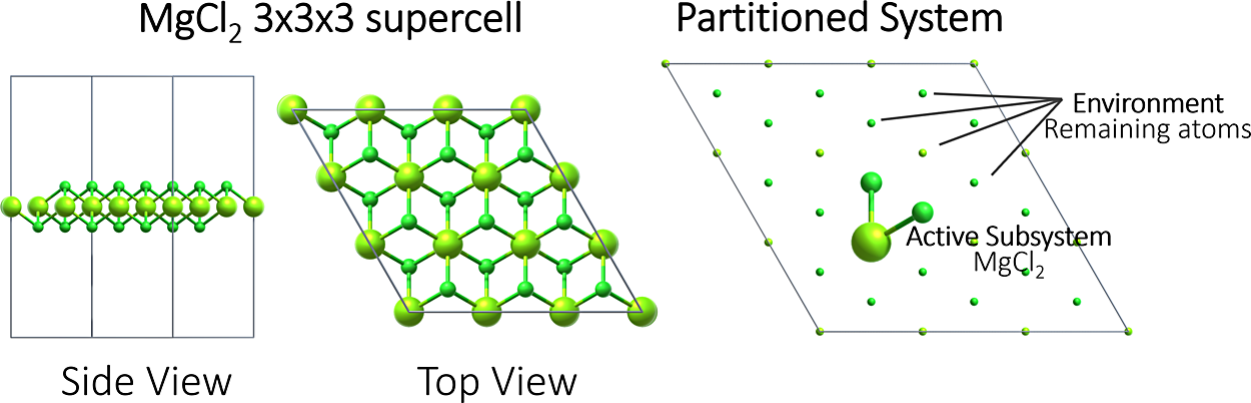
Visualization of the 3 × 3 surface supercell of MgCl_2_ and the partitioning into the active and environment subsystems. MgCl_2_ is considered as the molecular active subsystem and is shown with larger atoms, while the remaining smaller atoms constitute the periodic environment (light green: Mg and dark green: Cl).

Similarly, the optical gap of 2D MgCl_2_ is studied by constructing a 3 × 3 surface supercell of the unit cell taken from the computational 2D materials database (C2DB) database^91,92^ and considering the central MgCl_2_ cluster as the active subsystem (see Figure 3).

The excitation energies of all the embedded active subsystems (clusters) are evaluated using GW/BSE-in-DFT and CC2-in-DFT. Note: all the occupied and virtual orbitals are correlated. GW/BSE and CC2 excitation energies of the isolated clusters are also calculated to see the effect of embedding. In the case of 2D MgCl_2_, a 9 × 5 *k*-point grid is utilized for the total DFT density calculation, while in all other cases, a Γ-point DFT calculation is performed to obtain ρ^tot^. Note: Table S5 of the Supporting Information shows that increasing the *k*-mesh size has no effect on the CC2-in-DFT and GW/BE-in-DFT excitation energies, using LiF as an example.

In all cases, the Perdew–Burke–Ernzerhof (PBE) functional^93,94^ is used as the XC functional for the calculation of ρ^tot^ and ρ_iso_^act^, as well as for the evaluation of the nonadditive XC potential term in the embedding potential. The LC94 KEDF^95,96^ is utilized for the calculation of the naddKP term in the embedding potential. In the case of CC2-in-DFT embedding for 3D ionic solids, the dependence on the type of XC functional and KEDF is also studied by employing the LDA XC functional^97,98^ and Thomas-Fermi (TF)^99^ KEDF for XC and kinetic terms in the embedding potential, respectively.

The structures of all the systems are visualized using CrysX-3D Viewer^100^ and the corresponding crystallographic information files (CIFs) are provided in the Supporting Information.

## 4 Results and Discussion

### 4.1 Optical Gaps of Ionic Solids (3D)

**Figure 4:**
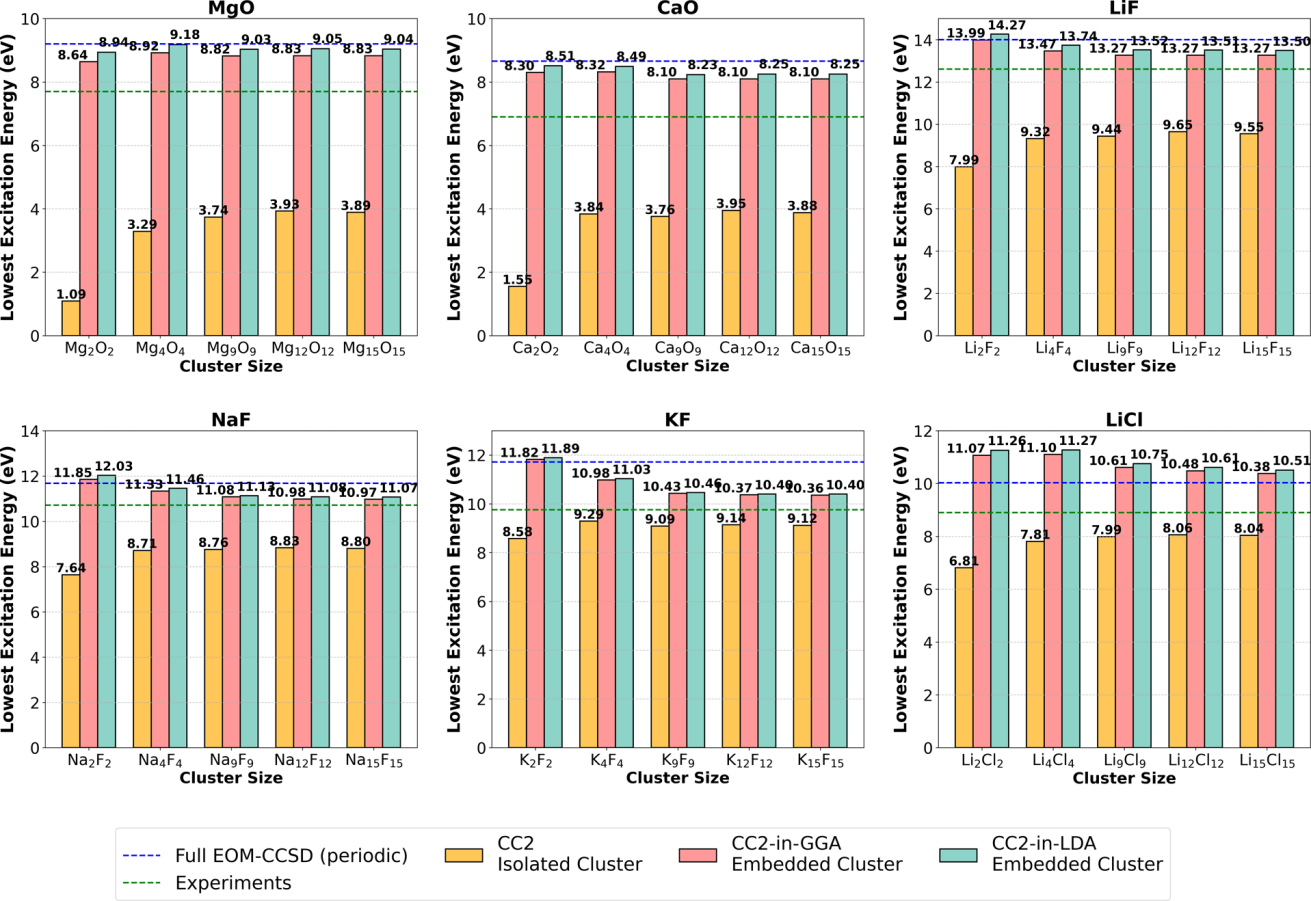
First singlet excitation energies (eV) of the different sized isolated and embedded clusters of various ionic solids from CC2 and CC2-in-DFT (DFT = LDA, GGA).

**Figure 5:**
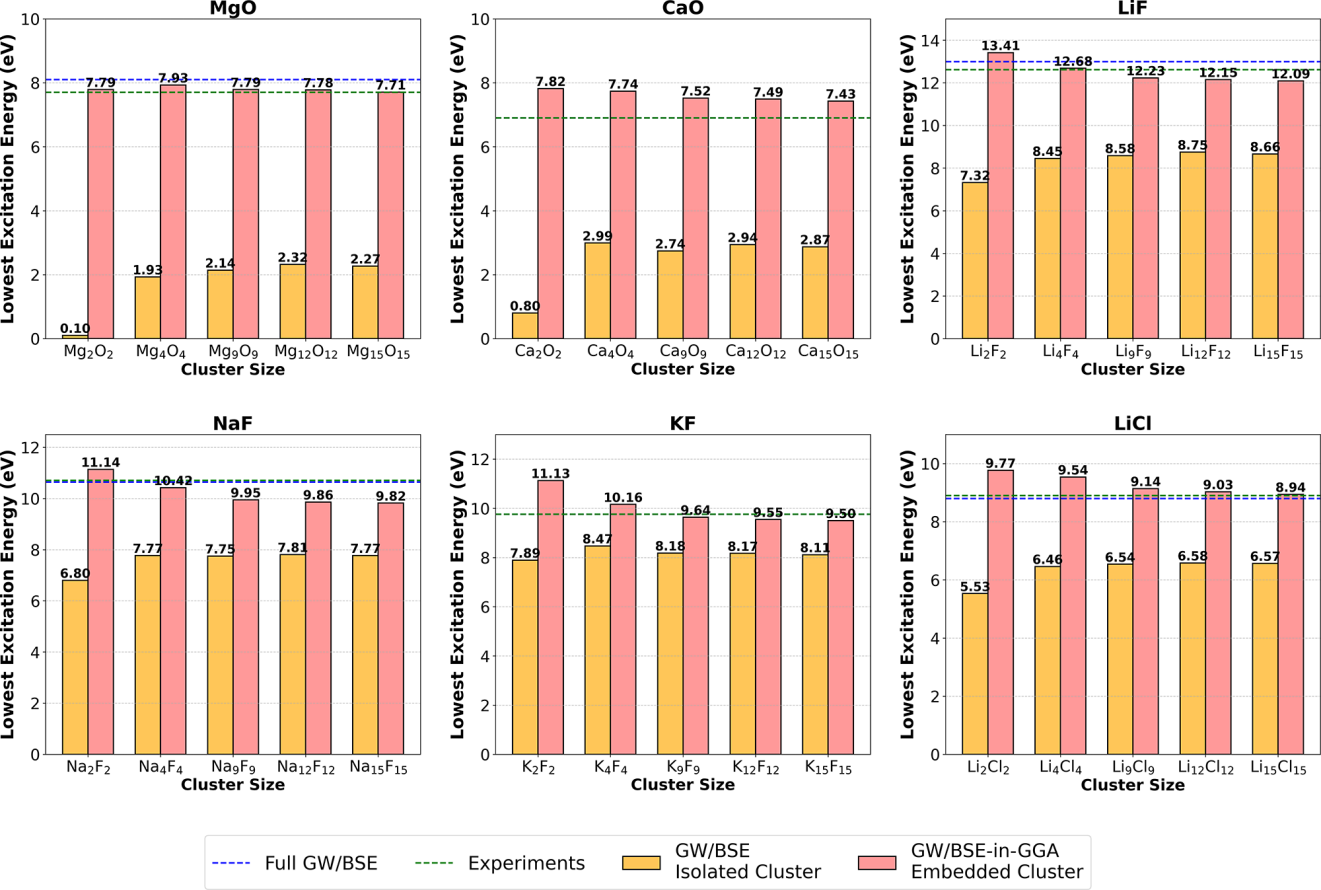
First singlet excitation energies (eV) of the different sized isolated and embedded clusters of various ionic solids from GW/BSE and GW/BSE-in-GGA.

Figure 4 shows the lowest-lying singlet excitation energies for different-sized clusters of the six solids considered in this work, calculated using CC2. The yellow bar corresponds to the CC2 excitation energy of the isolated (bare) cluster, which are calculated without embedding. The pink and teal bars correspond to the CC2-in-GGA and CC2-in-LDA excitation energies, respectively, of the embedded clusters. For CC2-in-GGA, both the XC functional and KEDF are of GGA type, while for CC2-in-LDA, they are of LDA type. Across all six materials, there is a notable difference between the isolated and embedded results, highlighting the influence of the embedding potential. The blue and green horizontal dashed-lines correspond to the optical gaps obtained from a full periodic EOM-CCSD calculation and reported experimental values, respectively. Figure 5 shows a similar bar graph as before but with GW/BSE as the high-level method, instead of CC2. GW/BSE-in-GGA results follow a similar trend as CC2-in-GGA results. The blue and green horizontal lines correspond to the reported values of optical gaps from periodic GW/BSE calculations and experiments, respectively. The results for all the materials are discussed in more detail below.

#### 4.1.1 MgO

**Figure 6:**
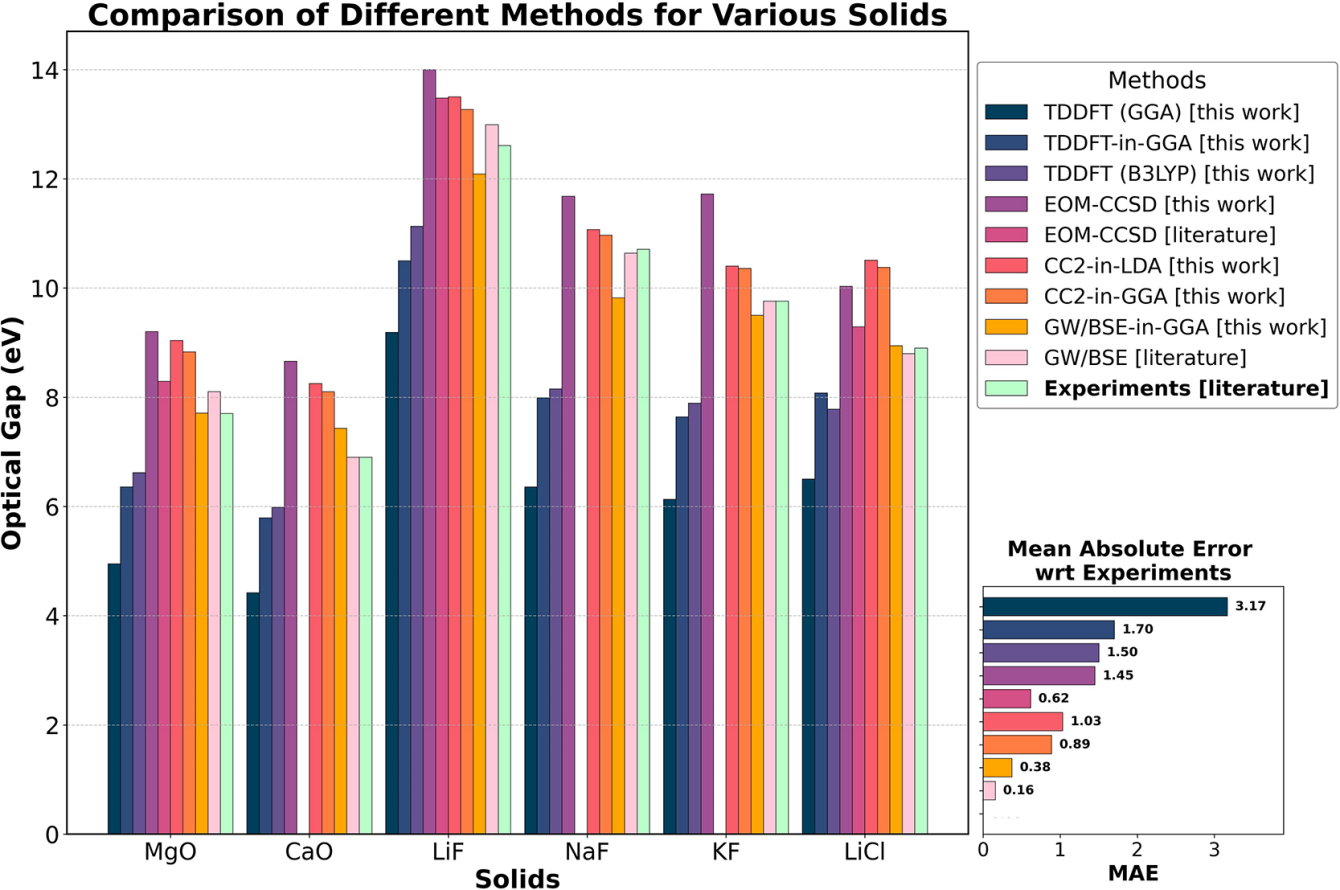
Lowest neutral singlet excitation energy (optical gap) of various materials obtained from cluster-in-periodic CC2-in-GGA, CC2-in-LDA, and GW/BSE-in-DFT embedding for the largest cluster sizes (this work) compared with the results from periodic EOM-CCSD (this work and literature), GW/BSE results, TDDFT (GGA) (this work), TDDFT (B3LYP) (this work), TDDFT-in-GGA (this work), and experimental results. Mean absolute errors (MAEs) of various methodologies are calculated with respect to experiments. EOM-CCSD literature values are from refs (27) and (29). GW/BSE literature values are from ref (102) (MgO), ref (82) (CaO), ref (87) (LiF, NaF, and KF), and ref (112) (LiCl). The experimental optical gaps are from ref (101) (MgO and CaO), ref (108) (LiF and NaF), ref (113) (KF), and ref (110) (LiCl).

For isolated MgO clusters, the CC2 (GW/BSE) excitation energy starts at a modest 1.09 eV (0.10 eV) for the smallest cluster Mg_2_O_2_ and goes to 3.89 eV (2.27 eV) for the largest Mg_15_O_15_. These values show a clear size dependence for the smaller sizes and are not completely converged. Furthermore, even the value corresponding to the largest cluster is significantly lower than the experimental value of 7.7 eV.^101^ In contrast, embedding the clusters within a periodic environment yields excitation energies remarkably consistent across different cluster sizes. The CC2-in-GGA values range from 8.64 to 8.92 eV, CC2-in-LDA values range from 8.94 to 9.18 eV, and GW/BSE-in-GGA values range from 7.71 to 7.93 eV, suggesting that the embedding effect remains relatively constant irrespective of the cluster size. Notably, these values are more than double those observed for isolated clusters, underlining the significant impact of the surrounding environment on the excitation energies for clusters of this size. Furthermore, the excitation energies obtained using the CC2-in-DFT method exhibit excellent convergence with respect to cluster size. The values show minimal variation (<0.01 eV) between Mg_9_O_9_ and Mg_15_O_15_ clusters, indicating convergence with respect to cluster size. Consequently, the excitation energy obtained for the largest cluster can be considered a reliable approximation of the optical gap predicted by the CC2-in-DFT approach. The GW/BSE-in-GGA excitation energies show a slightly slower convergence, changing by 0.07 eV when going from Mg_12_O_12_ to Mg_15_O_15_ cluster. Considering that the excitation energies of the embedded clusters are more or less converged with respect to the cluster size, the value corresponding to the largest cluster can be compared to various theoretical and experimental values in Figure 6. CC2-in-GGA yields a value (8.83 eV) that is slightly higher than the experimental optical gap of 7.7 by 1.13 eV. CC2-in-LDA predicts an even higher value of 9.05 eV. While CC2-in-DFT overestimates the experimental results, it demonstrates good agreement with the results obtained from periodic EOM-CCSD calculations: 8.29 eV in ref (27), 9.05 eV in ref (29), and 9.20 eV in this work. GW/BSE-in-DFT gives a value of 7.71 eV which is in remarkable agreement with the experimentally measured value and also in good agreement with the 8.1 eV value obtained from periodic GW/BSE calculations.^82,102^ The agreement of CC2-in-DFT and GW/BSE-in-DFT with EOM-CCSD and GW/BSE, respectively, strengthens the confidence in the embedding methodology.

Recently, Dittmer et al. examined the optical gaps of semiconductors by applying similarity-transformed EOM-CCSD (STEOM-CCSD) and embedded cluster method that utilizes point charges.^103^ However, their approach yields a value of 6.32 eV for MgO which is considerably lower than the experimental and other theoretical values.

Carter and co-workers have also investigated the excitation energy of MgO using various embedding methods, involving finite point charges, periodic point charges (PPC), and orbital-free DFT.^69^ Interestingly, they observed that DFT-based embedding underperformed compared to point charge embedding. For the Mg_4_O_4_ cluster, DFT-based embedding with multireference singles and doubles configuration interaction (MRSDCI) predicted an excitation energy of 5.63 eV, whereas PPC embedding with complete active space second-order perturbation theory (CASPT2) yielded an excitation energy of 6.81 eV. Moreover, the convergence of the excitation energy with respect to cluster size was slower compared to the present study.

For additional context, it is worth mentioning that DFT employing common nonhybrid functionals significantly underestimates the band gap of MgO, typically predicting values below 5 eV.^104^ In fact, periodic TDDFT with PBE XC functional predicts an excitation energy of 4.95 eV as can be seen in Figure 6. This substantial underestimation further underscores the crucial role of correlation effects in accurately describing the electronic properties of MgO. Even with a hybrid functional such as B3LYP,^98,105−107^ the predicted excitation is only 6.62 eV (see Figure 6), underestimating the experimental value by around 1.1 eV.

#### 4.1.2 CaO

Similar to MgO, the CC2 (GW/BSE) excitation energies of the isolated CaO clusters are quite small, ranging from 1.55 (0.80) to 3.88 eV (2.87 eV) for the smallest (Ca_2_O_2_) and largest (Ca_15_O_15_) cluster sizes, respectively, while the excitation energies of the embedded clusters show a significant increase (see Figures 4 and 5). In the case of CC2-in-GGA, the excitation energy is 8.30 eV for Ca_2_O_2_ and converges to 8.10 eV for clusters larger than Ca_9_O_9_. The CC2-in-LDA excitation energy corresponding to the smallest cluster is 8.51 eV and for the largest cluster (Ca_15_O_15_), it is 8.25 eV which is only 0.02 eV higher than the Ca_9_O_9_ cluster. The GW/BSE-in-GGA excitation energy corresponding to the smallest cluster is 7.82 eV and for the largest cluster (Ca_15_O_15_), it is 7.43 eV which can be considered to be converged within 0.06 eV. Similar to the trend observed for MgO, here also, the CC2-in-LDA excitation energy is slightly higher than the CC2-in-GGA excitation energy for all clusters, with the difference being in the range of 0.12–0.21 eV. The CC2-in-DFT and GW/BSE-in-DFT excitation energies of the largest clusters, which are converged with respect to cluster size, can be regarded as a reasonable approximation to the optical gap and are thus compared with other theoretical and experimental investigations presented in Figure 6. The CC2-in-GGA value of 8.10 eV and CC2-in-LDA value of 8.25 eV are in good agreement with the periodic EOM-CCSD value of 8.66 eV, calculated in this work. However, these values overestimate the 6.90 eV value obtained from GW/BSE and experiments.^82,101^ The magnitude of overestimation is similar to that observed in the case of MgO. The tendency of CC2-in-DFT and EOM-CCSD to overestimate the gap could be corrected by including triple excitations and considering finite-size effects as well as vibrational effects.^27,29^ On the other hand, GW/BSE-in-DFT value of 7.43 eV is in much better agreement with the 6.90 eV value reported from GW/BSE and experiments. Nonetheless, the accuracy of GW/BSE-in-DFT and CC2-in-DFT is quite impressive, especially considering their significantly lower computational cost compared to periodic GW/BSE and EOM-CCSD. Furthermore, as shown in Figure 6, the optical gaps obtained using TDDFT with PBE and B3LYP XC functionals are 4.42 and 5.98 eV, respectively, highlighting GW/BSE-in-DFT as the best performing and most efficient method.

#### 4.1.3 LiF

For LiF, the CC2 (GW/BSE) method predicts the first excitation energy for the smallest isolated cluster (Li_2_F_2_) to be 7.99 eV (7.32 eV), and for the largest isolated cluster (Li_15_F_15_), it is 9.55 eV (8.66 eV), as shown in Figures 4 and 5. These values are still quite distant from the experimentally measured values of 12.61^108^ and 12.80 eV,^109,110^ highlighting the role of environmental effects in this system. When using CC2-in-GGA, the excitation energy for the smallest embedded Li_2_F_2_ cluster is 13.99 eV and converges quickly to 13.27 eV for larger clusters (Li_9_F_9_, Li_12_F_12_, and Li_15_F_15_). In the case of CC2-in-LDA, the values for the smallest and largest clusters are 14.27 and 13.50 eV, respectively. The excitation energy from GW/BSE-in-GGA for the largest cluster Li_15_F_15_ is converged to within 0.06 eV. Clearly, CC2-in-DFT and GW/BSE-in-GGA values are converged with respect to cluster size and the values corresponding to the largest cluster can be considered as an approximation to the optical gap and compared with other theoretical and experimental values (see Figure 6). Similar to the case of MgO and CaO, CC2-in-GGA and CC2-in-LDA overestimate the experimental value (12.61 eV) slightly by 0.66 and 0.89 eV, respectively. The small difference of 0.23 eV between CC2-in-GGA and CC2-in-LDA indicates that both are able to capture the environmental effects with similar accuracy. CC2-in-DFT predictions are also in remarkable agreement with the 13.48 eV and similar values predicted by periodic EOM-CCSD calculations in literature and this work^27,29^ (see Figure 6). Furthermore, CC2-in-DFT values also compare very well with the optical band gap of 12.99 eV obtained from the GW/BSE approximation. GW/BSE-in-GGA predicts an optical gap of 12.09 eV which is 0.52 eV less than the experimental value but closer to it compared to CC2-in-GGA. Interestingly, CC2-in-DFT is closer to the periodic GW/BSE result than GW/BSE-in-GGA. Given the simplicity and cost-effectiveness of the embedding approach used, such good agreement with other state-of-the-art theoretical methods is encouraging, especially when compared to the fundamental gap of 9.19 eV obtained using the PBE XC functional.^111^ In fact, as can be seen in Figure 6, the optical gap obtained from TDDFT with GGA and B3LYP functionals is 9.19 and 11.13 eV with errors considerably larger than those obtained with CC2-in-DFT and GW/BSE-in-DFT. Therefore, DFT-based embedding effectively improves upon the suboptimal PBE functional used to construct the embedding potential and incorporates environmental effects and correlation successfully.

#### 4.1.4 NaF

Figures 4 and 5 show that the CC2 (GW/BSE) lowest singlet excitation energies of the smallest (Na_2_F_2_) and largest (Na_15_F_15_) isolated clusters are 7.64 eV (6.8 eV) and 8.80 eV (7.77 eV), respectively. When embedding is applied the excitation energy of the largest Na_15_F_15_ cluster increases to 10.97 eV for CC2-in-GGA, 11.07 eV for CC2-in-LDA, and 9.82 eV for GW/BSE-in-GGA. In all the cases the excitation energies are converged to within 0.04 eV when going from Na_12_F_12_ to Na_15_F_15_ cluster size. Considering the excitation energy of the largest embedded cluster as an approximation to the optical gap, it is compared to theoretical and experimental results in Figure 6. Values from both CC2-in-GGA (10.98 eV) and CC2-in-LDA (11.08 eV) are in excellent agreement with the measured value of 10.71 eV^108^ as well as the reported GW/BSE value of 10.64 eV.^87^ The periodic EOM-CCSD optical gap of 11.68 eV calculated in this work is also in reasonable agreement with the values from CC2-in-DFT with a slight overestimation. Interestingly, similar to LiF, the 9.82 eV value from GW/BSE-in-DFT is 0.82 eV lower than the reported periodic GW/BSE result. Therefore, for NaF, CC2-in-DFT performs better than GW/BSE-in-DFT, unlike the previous cases. Additionally, it is also worth noting that TDDFT (PBE) and TDDFT (B3LYP) yield a value of 7.99 and 8.15 eV, respectively, showcasing the superior performance of embedding methods.

#### 4.1.5 KF

For KF, unlike the previous cases, the excitation energies of the isolated clusters remain decently consistent across cluster sizes, for both CC2 and GW/BSE (see Figures 4 and 5). In fact, the CC2 excitation energy value corresponding to the largest cluster (9.12 eV) is quite close to the experimental value of 9.76 eV^113^ and reported periodic GW/BSE^87^ (see Figure 6). CC2-in-GGA predicts an excitation energy of 11.82 eV for the K_2_F_2_ cluster and 10.36 eV for the K_15_F_15_ cluster, showing a weaker environmental effect (1.24 eV for K_15_F_15_) compared to previously considered materials. CC2-in-LDA also predicts very similar values: 11.89 eV (K_2_F_2_) and 10.40 eV (K_15_F_15_). Both CC2-in-LDA and CC2-in-GGA excitation energies are converged with respect to cluster size as they do not change by more than 0.01 eV when going from K_12_F_12_ to K_15_F_15_ cluster. GW/BSE-in-GGA yields an excitation energy of 11.13 eV for the smallest cluster and 9.50 eV for the largest cluster. The convergence with respect to cluster size is slightly slow, with the GW/BSE-in-DFT excitation energy showing a decrease of 0.05 eV when going from K_12_F_12_ to K_15_F_15_. Once again, the CC2-in-DFT and GW/BSE-in-DFT excitation energy corresponding to the largest cluster is compared to other theoretical methods and experimental results in Figure 6. CC2-in-GGA and CC2-in-LDA overestimate the experimental value of 9.76 by 0.60 and 0.64 eV, respectively. Interestingly, CC2 for the isolated K_15_F_15_ underestimates the experimental value by a similar amount of 0.62 eV. The GW/BSE-in-DFT excitation energy on the other hand, is in excellent agreement with the experimental value of 9.76 eV with an error of just 0.26 eV. Therefore, both CC2-in-DFT and GW/BSE-in-DFT perform better than TDDFT with B3LYP functional which predicts a gap of 7.89 eV, underestimating the experimental value by 1.87 eV.

#### 4.1.6 LiCl

For LiCl, the CC2 excitation energy of the smallest cluster is 6.81 eV for the isolated case, which increases to 11.07 eV for CC2-in-GGA and 11.89 eV for CC2-in-LDA (see Figure 4). Similarly, the GW/BSE and GW/BSE-in-DFT excitation energies for the smallest cluster are 5.53 and 9.77 eV, respectively. For the largest cluster, the CC2 excitation energy for the isolated case is 8.04 eV which increases by more than 2 eV for the embedded case: 10.48 eV with CC2-in-GGA and 10.61 eV with CC2-in-LDA. Analogously, the GW/BSE excitation energy of the largest cluster is 6.57 eV, which increases by more than 2 eV, reaching 8.94 eV with embedding. Compared to all the previous cases, the CC2-in-DFT and GW/BSE-in-DFT excitation energies exhibit the slowest convergence for LiCl, changing by about 0.1 eV when increasing the cluster size from Li_12_Cl_12_ to Li_15_Cl_15_. As observed for all other materials, the CC2-in-LDA excitation energy is slightly higher than the CC2-in-GGA value by 0.13 eV. The CC2-in-DFT and GW/BSE-in-DFT excitation energies corresponding to the largest cluster are considered as an approximation to the optical gap and compared to values from other theories as well as the measured value in Figure 6. Similar to the trend observed in all other materials, the CC2-in-DFT excitation energies overestimate the measured value of 8.90 eV^110^ by 1.48 eV in the case of CC2-in-GGA and 1.61 eV with CC2-in-LDA. Therefore, among all materials CC2-in-DFT exhibits the largest error with respect to the measured value for LiCl. The CC2-in-DFT excitation energies are also higher than the 9.29 eV value obtained using periodic EOM-CCSD in ref (27) by about 1.2 eV but compare well with the EOM-CCSD value of 10.03 eV calculated in the present work. GW/BSE-in-DFT excitation energy (8.94 eV), however, is in excellent agreement with the experimental value of 8.90 eV as well as the periodic GW/BSE value of 8.80 eV.^112^ It is also worth mentioning that for LiCl, TDDFT (B3LYP) predicts an excitation energy of 7.78 eV, which is better than CC2-in-DFT results.

#### 4.1.7 Additional Insights

The behavior of excitation energies in embedded clusters reveals some intriguing trends. Specifically, when examining embedded clusters, a decrease in excitation energies is observed when moving from smaller to larger clusters. In contrast, for isolated clusters, there is a slight increase in excitation energies when transitioning from A_9_X_9_ to A_12_X_12_, followed by a decrease when moving to A_15_X_15_. This lack of a clear trend can be attributed to the varying shapes of the clusters, which result in inconsistent contributions from surface and edge-localized states (see ref (114)).

One of the key advantages of DFT-based embedding is its ability to suppress these surface and edge effects effectively, leading to more consistent and reliable results. As shown in Figure 6, the CC2-in-GGA and CC2-in-LDA approaches yield mean absolute errors (MAEs) of 0.89 and 1.03 eV, respectively, when compared to experimental data. In contrast, the GW/BSE-in-GGA method achieves a significantly lower MAE of 0.38 eV, indicating superior accuracy. Consequently, both CC2-in-DFT and GW/BSE-in-DFT outperform the TDDFT (B3LYP) approach (with an MAE of 1.5 eV) in terms of accuracy. Interestingly, periodic EOM-CCSD calculations from this work have a large MAE of 1.45 eV with respect to experiments. This could be because first, no correction is applied to make up for the relatively high number of frozen orbitals employed in the calculations. Second, the EOM-CCSD calculations converge very slowly with the number of *k*-points, so a 3 × 3 × 3 *k*-mesh may be far from convergence. These errors can be significant as seen in refs (27) and (29).

When comparing the convergence properties of different methods, CC2-in-DFT stands out, converging faster than both GW/BSE-in-DFT with respect to cluster size. Furthermore, CC2 excitation energies are consistently higher than those obtained from GW/BSE, highlighting a systematic difference between the two methods.

An interesting trend emerges when comparing GW/BSE-in-DFT with periodic GW/BSE: the discrepancy between the two methods diminishes as we move from LiF to NaF to KF. This observation suggests that as the ionic character of the solid increases, the accuracy of the KEDF becomes less of a limiting factor. Supporting this, the difference between CC2-in-LDA and CC2-in-GGA excitation energies decreases from 0.23 eV for LiF to just 0.04 eV for KF.

Since, the MAE of CC2-in-DFT has been found to be slightly higher than GW/BSE-in-DFT, it is important to mention some of the sources of error that can influence the accuracy of CC2-in-DFT calculations. One significant source is the potential discrepancy between the DFT and WFT (CC2) descriptions; since the embedding potential is derived from DFT, it may carry inherent inaccuracies into the WFT calculations. Additionally, approximations arise in the estimation of the embedding potential itself, as the active and environment subsystem densities are not exact, even at the DFT level. The choice of kinetic energy density functional (KEDF) further affects the results, introducing variability depending on the selected functional. It is also essential to acknowledge that CC2, despite being a high-level method, is not guaranteed to reproduce experimental results precisely. The use of a monomolecular basis set in embedding calculations is an additional approximation. These factors collectively contribute to the potential sources of error in CC2-in-DFT, necessitating careful consideration when interpreting results.

To further explore the sources of error, TDDFT-in-GGA calculations are performed. These calculations show that TDDFT-in-GGA tends to overestimate the reference periodic TDDFT (GGA) result by an average of 1.46 eV. This could be attributed to the simplistic nature of approximating the environment density as described in Section 2.1.3. The overestimation might explain why CC2-in-DFT sometimes overestimates the optical gap compared to experimental results. However, when compared to the periodic EOM-CCSD results presented in this work, CC2-in-DFT actually underestimates the EOM-CCSD values, with an MAE of 0.68 eV. This suggests the presence of some beneficial error cancellation, possibly due to the absence of the first-order correction term discussed in Section 2.1.1. For a deeper understanding of the error sources in WFT-in-DFT methods, readers are encouraged to consult ref (64).

#### 4.1.8 Computational Performance

**Figure 7:**
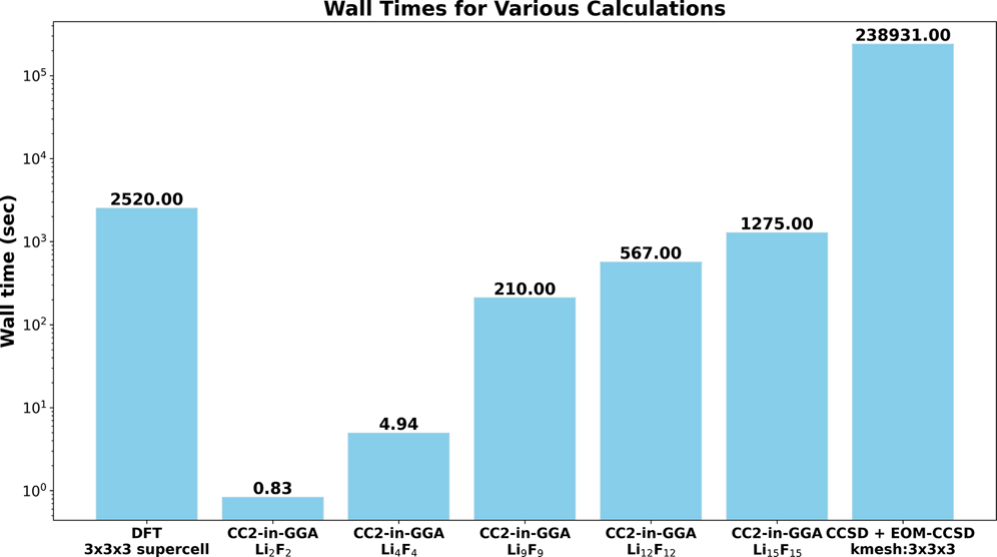
Wall timings (in seconds) of various calculations: DFT calculation of the supercell, CC2-in-DFT calculations for various cluster sizes, and periodic EOM-CCSD calculation.

It is also worth highlighting the computational performance of the current CC2-in-DFT and GW/BSE-in-DFT approaches. The majority of the time for the embedding potential calculation is consumed by the total periodic DFT calculation, which is necessary for the KEDF-based embedding method outlined in Section 2.1.3. As an illustrative example, the wall times for LiF are considered. Figure 7 shows the wall times in seconds for the DFT calculation on the total system, CC2-in-DFT calculations for various LiF clusters, and a periodic EOM-CCSD calculation. All timings are obtained using 16 CPU cores. The DFT calculation for the total system to generate ρ^tot^ takes about 2520 s or 42 min taking advantage of the linear scaling acceleration techniques implemented in TURBOMOLE.^56−58^ The CC2-in-DFT excitation energy calculation for the largest Li_15_F_15_ cluster required another 1275 s or 21.25 min. Therefore, a complete CC2-in-DFT calculation takes around 63 min in total, while a periodic EOM-CCSD calculation, with 16 occupied and 16 virtual orbitals correlated, took about 2.4 × 10^5^ s or 2.76 days. This shows that the computational cost of the CC2-in-DFT method is negligible compared to periodic EOM-CCSD.

**Figure 8:**
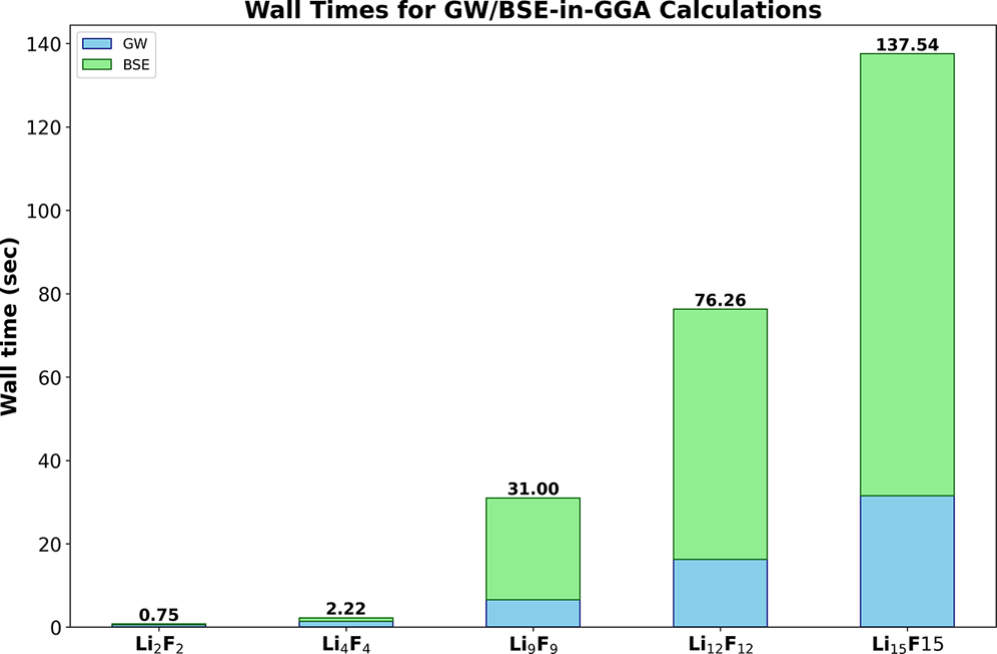
Wall timings (s) of GW/BSE-in-DFT calculations for various cluster sizes.

Additionally, the wall timings for the GW/BSE-in-DFT calculations are also presented in Figure 8. The largest Li_15_F_15_ cluster requires only about 138 s for obtaining the single-shot G_0_W_0_ quasiparticle energies as well as the excitation energies from BSE. This shows that while being the most accurate of the embedding methods considered in this work, GW/BSE-in-DFT is also the most computationally efficient.

### 4.2 Oxygen Vacancy in MgO

As another application of cluster-in-periodic embedding, the excitation energy of the oxygen vacancy defect in MgO (neutral *F*-center) is also investigated. The embedding potential is constructed using the PBE XC functional and the LC94 KEDF. The excitation energy of Mg_4_O_4_ cluster with an oxygen vacancy at the center (see Figure 3), as calculated using CC2-in-DFT and GW/BSE-in-DFT is 6.25 and 5.30 eV, respectively. The CC2-in-DFT result agrees well with the value of 6.26 eV^115^ reported recently using density matrix embedding theory (DMET) and complete active space self-consistent field (CASSCF), however, it overestimates the measured value of 5 eV.^116,117^ This overestimation is not unexpected as even for the bulk case, CC2-in-DFT overestimated the experimental value by a similar magnitude (∼1.1 eV). The CC2-in-DFT value (6.26 eV) is also in reasonable agreement with the reported CCSD values of 5.31^118^ and 5.28 eV,^119^ especially considering the fact that unlike ref (119), the bulk geometry is not reoptimized after introducing the defect, which could also introduce further changes. On the other hand, GW/BSE-in-DFT result of 5.30 eV is in excellent agreement with the experimental value, which is expected as it also worked well for the MgO 3D solid. Therefore, both CC2-in-DFT and GW/BSE-in-DFT seem to be working well for studying the excitation energies of defects as well.

### 4.3 Optical Gap of 2D MgCl_2_

As a final test and application of the CC2-in-DFT and GW/BSE-in-DFT methods for cluster-in-periodic embedding, the optical gap of 2D MgCl_2_ is investigated. This time only the smallest possible MgCl_2_ cluster, i.e., one formula unit, is carved out from the MgCl_2_ supercell. Only the GGA type XC functional and KEDF are used for constructing the embedding potential. The lowest excitation energy from CC2-in-DFT is 8.03 eV while from GW/BSE-in-DFT is 6.76 eV. The fundamental band gap from single shot G_0_W_0_ calculation, as reported in the C2DB database,^91,92^ is 9.599 eV.^120^ The exciton binding energy from BSE is 1.86 eV,^120^ implying an optical gap of 7.74 eV. Therefore, the CC2-in-DFT value (8.03 eV) is in good agreement with the reference GW/BSE result, however, the GW/BSE-in-DFT result underestimates the reference by about 1 eV. This is similar to the case of LiF and NaF, where GW/BSE-in-DFT underestimated the optical gap by close to 1 eV when compared to periodic GW/BSE calculations. In fact, it has been an overall trend that GW/BSE excitation energies are in general 1 eV lower than CC2 values, with or without embedding. So, wherever CC2-in-DFT is closer to the reference values, GW/BSE-in-DFT would underestimate the reference.

## 5 Conclusions and Outlook

An implementation of cluster-in-periodic embedding is presented for the calculation of optical properties such as optical gaps and defect excitation energies in materials with ionic bonding. The lowest-lying excitation energy of the molecule-like active subsystem (cluster) is calculated using CC2 as well as GW/BSE in the presence of a periodic environment whose effect is captured by the KEDF-based embedding potential and used to approximate the optical gap. It is shown that GW/BSE-in-DFT and CC2-in-DFT offer a practical and efficient alternative to more expensive and complex methods such as periodic EOM-CCSD and GW/BSE and capture the correlation effects at a fraction of the computational cost.

For 3D ionic solids, both GW/BSE-in-DFT and CC2-in-DFT excitation energies of the clusters carved out from the bulk show a significant shift in the presence of the embedding potential, especially for the oxides (MgO and CaO), underscoring the environmental effects. Furthermore, in most cases, the excitation energy of the embedded cluster quickly converges with cluster size compared to the excitation energy of the isolated cluster. Interestingly, while the CC2 or GW/BSE excitation energy of the isolated clusters increases with cluster size, the embedded excitation energy starts from a higher value and converges to a lower value. In all the cases, CC2-in-DFT overestimates the experimental optical gap values but is in good agreement with EOM-CCSD results, though the latter may not be converged due to computational constraints. On the other hand, GW/BSE-in-DFT results compare excellently to both experimental and reference periodic GW/BSE values. The dependence of CC2-in-DFT results on the functional type was also investigated by using both LDA and GGA type XC functionals and KEDFs. It was found that CC2-in-LDA values are consistently slightly higher (0.15 eV on average) than the CC2-in-GGA values.

The embedding strategy is also found to perform well in the study of defects as demonstrated by the study of the excitation energy of the O vacancy in MgO. Although CC2-in-DFT again slightly overestimates the excitation energy compared to experimental results, it accurately captures the energy shift from the pure to the defective state. Specifically, CC2-in-DFT predicts a shift of 2.57 eV, which is remarkably close to the experimental value of 2.7 eV. Similar to the case of ionic solids, GW/BSE-in-DFT excitation energy of the defect is much closer to the experimental value with an error of jut 0.30 eV.

Finally, CC2-in-DFT is found to predict the optical gap of 2D MgCl_2_ quite accurately compared to the GW/BSE results, despite the use of just one formula-unit as the cluster for the active subsystem. In contrast to earlier observations, GW/BSE-in-DFT is found to underestimate the reference result and performs worse than CC2-in-DFT.

The performance of DFT-based embedding, especially GW/BSE-in-DFT, is quite impressive considering the use of an approximate KEDF-based embedding potential and the simplistic method for the construction of the environment density and in turn the embedding potential, which may not fulfill the *v*_s_—representability condition for the subsystem densities. This is not surprising, as the dominant interactions between subsystems are electrostatic in nature for the ionic materials considered in this work. The electrostatic interactions are handled exactly in the KEDF-based embedding potential, with the primary issue being the choice of environmental density, likely the major source of errors in this study. Lastly, the excitation energy calculation in this work does not include the first-order correction due to the embedding potential, which may be important for WFT-in-DFT calculations. Interestingly, GW/BSE-in-DFT results benefit from fortuitous error cancellation resulting in good performance.

Future work may explore the use of more accurate density partitioning as well as the use of higher-level hybrid XC functionals for the treatment of the environment. The present work can also be extended to calculate the absorption spectra of the ionic materials such as metal halide perovskites. Due to the above-mentioned approximations, this method cannot be used for systems with covalent bonds. Therefore, the authors are currently exploring a projection-based periodic-in-periodic (*k*WFT-in-*k*DFT) embedding approach where both the subsystems are treated with periodic boundary conditions, but the active subsystem is treated with periodic EOM-CCSD (*k*WFT) and the environment with periodic DFT (*k*DFT).

## Supporting Information Available

Tables corresponding to values reported in Figures 4–6. Wall times of periodic EOM-CCSD calculations. CIF files containing the coordinates of the supercells of MgO, CaO, LiF, NaF, KF, LiCl, MgCl_2_, and MgO with O vacancy utilized in this work (ZIP)
