# Supplementary material for: Optical Gaps of Ionic Materials from GW/BSE-in-DFT and CC2-in-DFT
Source: J Chem Theory Comput. 2024 Oct 17;20(21):9592–605. doi: 10.1021/acs.jctc.4c00819 (PMC11562370; doi:10.1021/acs.jctc.4c00819)
Supplement: Supplementary file 1 — ct4c00819_si_001.zip [file ct4c00819_si_001.zip › Supporting_Information/Supporting_Information.pdf]

Supporting Information: Optical Gaps of Ionic Materials from GW/BSE-in-DFT and CC2-in-DFT

Manas Sharma[†,‡] and Marek Sierka[*,†]

†*Otto Schott Institute of Materials Research, Friedrich Schiller Unversity Jena, Löbdergraben 32, 07743 Jena, Germany*
‡Present address: *Department of Chemical Engineering, Indian Institute of Science, Bengaluru, Karnataka 560012, India*

E-mail: marek.sierka@uni-jena.de

1 Cluster Size Dependence of CC2, CC2-in-LDA, and CC2-in-GGA

Table S1: First singlet excitation energies (eV) of the different sized isolated and embedded clusters of various ionic solids from CC2 and CC2-in-DFT (DFT = LDA, GGA)

Cluster	CC2<br>Isolated Cluster	CC2-in-GGA<br>Embedded Cluster	CC2-in-LDA<br>Embedded Cluster
		**MgO**	
Mg_2O_2	1.09	8.64	8.94
Mg_4O_4	3.29	8.92	9.18
Mg_9O_9	3.74	8.82	9.03
$Mg_{12}O_{12}$	3.93	8.83	9.05
$Mg_{15}O_{15}$	3.89	8.83	9.04
		**CaO**	
Ca_2O_2	1.55	8.30	8.51
Ca_4O_4	3.84	8.32	8.49
Ca_9O_9	3.76	8.10	8.23
$Ca_{12}O_{12}$	3.95	8.10	8.25
$Ca_{15}O_{15}$	3.88	8.10	8.25
		**LiF**	
Li_2F_2	7.99	13.99	14.27
Li_4F_4	9.32	13.47	13.74
Li_9F_9	9.44	13.27	13.52
$Li_{12}F_{12}$	9.65	13.27	13.51
$Li_{15}F_{15}$	9.55	13.27	13.50
		**NaF**	
Na_2F_2	7.64	11.85	12.03
Na_4F_4	8.71	11.33	11.46
Na_9F_9	8.76	11.08	11.13
$Na_{12}F_{12}$	8.83	10.98	11.08
$Na_{15}F_{15}$	8.80	10.97	11.07
		**KF**	
K_2F_2	8.58	11.82	11.89
K_4F_4	9.29	10.98	11.03
K_9F_9	9.09	10.43	10.46
$K_{12}F_{12}$	9.14	10.37	10.40
$K_{15}F_{15}$	9.12	10.36	10.40
		**LiCl**	
Li_2Cl_2	6.81	11.07	11.26
Li_4Cl_4	7.81	11.10	11.27
Li_9Cl_9	7.99	10.61	10.75
$Li_{12}Cl_{12}$	8.06	10.48	10.61
$Li_{15}Cl_{15}$	8.04	10.38	10.51

2 Cluster Size Dependence of GW/BSE and GW/BSE-in-GGA

Table S2: First singlet excitation energies (eV) of the different sized isolated and embedded clusters of various ionic solids from GW/BSE and GW/BSE-in-GGA

Cluster	GW/BSE Isolated Cluster	GW/BSE-in-GGA Embedded Cluster
	**MgO**	
Mg_2O_2	0.10	7.79
Mg_4O_4	1.93	7.93
Mg_9O_9	2.14	7.79
$Mg_{12}O_{12}$	2.32	7.78
$Mg_{15}O_{15}$	2.27	7.71
	**CaO**	
Ca_2O_2	0.80	7.82
Ca_4O_4	2.99	7.74
Ca_9O_9	2.74	7.52
$Ca_{12}O_{12}$	2.94	7.49
$Ca_{15}O_{15}$	2.87	7.43
	**LiF**	
Li_2F_2	7.32	13.41
Li_4F_4	8.45	12.68
Li_9F_9	8.58	12.23
$Li_{12}F_{12}$	8.75	12.15
$Li_{15}F_{15}$	8.66	12.09
	**NaF**	
Na_2F_2	6.80	11.14
Na_4F_4	7.77	10.42
Na_9F_9	7.75	9.95
$Na_{12}F_{12}$	7.81	9.86
$Na_{15}F_{15}$	7.77	9.82
	**KF**	
K_2F_2	7.89	11.13
K_4F_4	8.47	10.16
K_9F_9	8.18	9.64
$K_{12}F_{12}$	8.17	9.55
$K_{15}F_{15}$	8.11	9.50
	**LiCl**	
Li_2Cl_2	5.53	9.77
Li_4Cl_4	6.46	9.54
Li_9Cl_9	6.54	9.14
$Li_{12}Cl_{12}$	6.58	9.03
$Li_{15}Cl_{15}$	6.57	8.94

3 Comparison of Different Methods

Table S3: Lowest neutral singlet excitation energy or optical gap (eV) of various materials obtained from different methods compared with experimental results. Mean absolute errors (MAE) and mean squared errors (MSE) are calculated with respect to experiments. The TDDFT-in-GGA, CC2-in-GGA, CC2-in-LDA, and GW/BSE-in-GGA results are for the largest cluster size.

Solid	TDDFT (GGA) (this work)	TDDFT-in-GGA (this work)	TDDFT (B3LYP) (this work)	EOM-CCSD (this work)	EOM-CCSD	CC2-in-GGA (this work)	CC2-in-LDA (this work)	GW+BSE	GW/BSE-in-GGA (this work)	Experiments
MgO	4.95	6.36	6.62	9.20	8.29	8.83	9.04	8.10	7.71	7.7
CaO	4.42	5.79	5.98	8.66	–	8.10	8.25	6.90	7.43	6.9
LiF	9.19	10.50	11.13	14.00	13.48	13.27	13.50	12.99	12.09	12.61
NaF	6.36	7.99	8.15	11.68	–	10.97	11.07	10.64	9.82	10.71
KF	6.13	7.64	7.89	11.72	–	10.36	10.40	9.76	9.50	9.76
LiCl	6.50	8.08	7.78	10.03	9.29	10.38	10.51	8.80	8.94	8.9
**MAE**	3.17	1.70	1.50	1.45	0.62	0.89	1.03	0.16	0.38	0
**MSE**	10.54	3.34	2.58	2.22	0.41	0.96	1.26	0.05	0.23	0

4 Wall Timings for Periodic EOM-CCSD Calculations using PySCF

Table S4: Wall Timings (seconds) for periodic EOM-CCSD Calculations using PySCF with different numbers of k-points. The system had 16 CPU cores. The table includes timings for Hartree-Fock (HF), CCSD, and EOM-CCSD calculations.

k-mesh	HF (s)	CCSD (s)	EOM-CCSD (s)
1x1x1	4	1	1
2x1x1	6	8	13
2x2x1	14	77	112
2x2x2	32	837	1400
3x3x1	45	1263	1960
3x3x3	335	91563	147368

5 *k*-mesh Size Dependence of CC2-in-GGA and GW/BSE-in-GGA Excitation Energies of LiF Clusters

Table S5: Excitation energies for different sized LiF clusters and *k*-mesh sizes using CC2-in-GGA and GW/BSE-in-GGA methods.

Cluster	1×1×1		2×2×2		3×3×3	
	CC2-in-GGA	GW/BSE-in-GGA	CC2-in-GGA	GW/BSE-in-GGA	CC2-in-GGA	GW/BSE-in-GGA
Li_9F_9	13.27	12.23	13.27	12.23	13.27	12.23
$Li_{12}F_{12}$	13.27	12.15	13.27	12.15	13.27	12.15
$Li_{15}F_{15}$	13.27	12.09	13.27	12.09	13.27	12.09
